# Efficacy of repetitive transcranial magnetic stimulation with different application parameters for post-stroke cognitive impairment: a systematic review

**DOI:** 10.3389/fnins.2024.1309736

**Published:** 2024-03-19

**Authors:** Yuhan Wang, Linjia Wang, Xixiu Ni, Minjiao Jiang, Ling Zhao

**Affiliations:** ^1^Acupuncture and Moxibustion College, Chengdu University of Traditional Chinese Medicine, Chengdu, Sichuan, China; ^2^Acupuncture and Moxibustion College, Nanjing University of Traditional Chinese Medicine, Nanjing, China; ^3^Acupuncture Clinical Research Center of Sichuan Province, Chengdu, China; ^4^Key Laboratory of Acupuncture for Senile Disease (Chengdu University of TCM), Ministry of Education, Chengdu, China

**Keywords:** stroke, cognitive impairment, repetitive transcranial magnetic stimulation, application parameters, randomized controlled trials

## Abstract

**Background:**

Cognitive impairment is a prevalent consequence of stroke, seriously affecting recovery and quality of life while imposing substantial burdens on both patients’ families and society. Repetitive transcranial magnetic stimulation (rTMS) has emerged as an effective intervention for post-stroke cognitive impairment (PSCI). However, the a lack of standardized and explicit guidelines regarding rTMS application parameters. Therefore, this study systematically evaluated the efficacy of various parameters of rTMS in treating PSCI and explored its potential mechanism.

**Methods:**

We conducted a comprehensive search across seven scientific databases, namely China National Knowledge Infrastructure (CNKI), Wanfang Data Knowledge Service Platform (Wanfang), China Science and Technology Journal Database (VIP), Web of Science, PubMed, Embase, and Cochrane Library, to identify randomized controlled trials (RCTs) investigating the efficacy of rTMS for PSCI. The search encompassed the period from database creation until July 28, 2023. To evaluate the risk of bias in included studies, we employed the Cochrane recommended risk of bias assessment tool. Furthermore, we extracted relevant clinical application parameters associated with rTMS and performed comparative analyses to assess their therapeutic effects under different parameter settings.

**Results:**

The present study included 45 RCTs involving a total of 3,066 patients with PSCI. Both high-frequency repetitive transcranial magnetic stimulation (HF-rTMS) and low-frequency repetitive transcranial magnetic stimulation (LF-rTMS) demonstrated safety and efficacy, yet failed to exhibit significant differentiation in terms of cognitive improvement. Furthermore, intermittent theta burst stimulation (iTBS), although yielding positive results, did not surpass traditional rTMS in effectiveness. Combining HF-rTMS with LF-rTMS resulted in superior efficacy compared to single rTMS intervention. Moreover, the combination of rTMS with other cognitive therapies exhibited potential for enhanced benefits among patients.

**Conclusion:**

rTMS can effectively and safely enhance cognitive function, improve quality of life, and enhance activities of daily living in patients with PSCI. Furthermore, the combination of rTMS with other conventional rehabilitation methods can yield additional positive effects. However, due to insufficient evidence, an optimal parameter protocol for rTMS can not be currently recommended. Future research should prioritize orthogonal experimental design methods that incorporate multiple parameters and levels to determine the optimal parameter protocol for rTMS in PSCI.

## 1 Introduction

The high morbidity, disability, and mortality rates associated with stroke have positioned it as the second leading cause of death globally and the primary cause of disability (Naghavi et al., 2017; Vos et al., 2020). Consequently, stroke has emerged as a critical public health concern worldwide (Tsao et al., 2023). Post-stroke cognitive impairment (PSCI), a common complication following stroke, is characterized by cognitive deficits that persist for 3 to 6 months after the event (Wang et al., 2021a). The prevalence of PSCI ranges from 24 to 53.4% among stroke patients (Douiri et al., 2013; Lo et al., 2019), with an increasing incidence trend observed (Huang et al., 2022). PSCI significantly impacts patients’ quality of life, activities of daily living, and post-stroke survival rate (Rohde et al., 2019; Wang et al., 2021a).

For PSCI, the primary treatment modalities encompass pharmacotherapy and traditional non-pharmacological rehabilitation approaches, such as cognitive training. Pharmacotherapy options include cholinesterase inhibitors (e.g., donepezil) and N-methyl-D-aspartate receptor antagonists (e.g., carpalatin) (Gorelick et al., 2011; Kim et al., 2020). Although these drugs can enhance patients’ cognitive function, they are associated with various adverse reactions, including diarrhea, nausea, insomnia, and even psychiatric symptoms like irritability and aggressive behavior (Farooq et al., 2017; Sun, 2018; Loetscher et al., 2019). However, the efficacy of traditional non-pharmacological cognitive rehabilitation training in PSCI remains limited due to prolonged intervention duration and suboptimal patient compliance, thus necessitating further exploration (Merriman et al., 2019).

Repetitive transcranial magnetic stimulation (rTMS) represents a highly promising non-invasive brain stimulation technique that can modulate cerebral cortex excitability in a non-invasive manner. By inducing enduring changes in neural plasticity through magnetic fields and reorganizing functional connectivity between specific regions, it holds the potential for enhancing brain network functionality and facilitating recovery of cognitive function among stroke patients (Hernandez-Pavon and Harvey, 2019).

Although numerous randomized controlled trials (RCTs) (Duan and Ding, 2016; Chen et al., 2017, 2019; Ding et al., 2019) have demonstrated the effective enhancement of cognitive function, daily living abilities, and quality of life in stroke patients through rTMS, there remains a lack of standardized and clear application parameters for rTMS, including intensity, frequency, and treatment duration. These parameters are crucial factors that influence the clinical outcomes. Therefore, this study aims to analyze recent RCTs investigating rTMS treatment for PSCI, evaluate the efficacy of various rTMS parameters on PSCI, and explore potential mechanisms of action. We hope that this study will offer valuable guidance and evidence-based medicine support for the clinical implementation of rTMS in the management of PSCI.

## 2 Methods

The protocol has been registered with PROSPERO (Registration number: CRD42023460450).

### 2.1 Inclusion criteria

The studies meeting the following criteria were included:

#### 2.1.1 Types of studies

The scope of this study was confined to RCTs investigating the efficacy of rTMS in patients diagnosed with PSCI.

#### 2.1.2 Population

The inclusion criteria were defined as follows: (1) all patients with ischemic or hemorrhagic stroke exhibited evident imaging pathological evidence on magnetic resonance imaging (MRI) or computed tomography (CT); (2) patients diagnosed with PSCI through clinical examination; (3) patients without any neurological disorders, including Parkinson’s disease, Alzheimer’s disease, or other causes of cognitive impairment.

#### 2.1.3 Intervention and comparison

The intervention group was administered rTMS, while the control group received either standard rehabilitation or sham/placebo rTMS. Both groups were provided with the same standard care.

#### 2.1.4 Outcome

Outcome measures included at least one of the following: The study considered one or more objective outcome indicators such as Montreal Cognitive Assessment Scale (MoCA), Mini-Mental State Examination (MMSE), Loewenstein Occupational Therapy Cognitive Assessment (LOTCA), the Tower of London Test (TOLT).

### 2.2 Exclusion criteria

We excluded literature from the search: (1) non-RCTs, such as case reports, reviews, and animal experiments; (2) baseline consistency tests were not given; (3) data were incomplete or full text was unavailable; (4) duplicate articles were published.

### 2.3 Data sources and search strategy

We searched seven scientific databases: China National Knowledge Infrastructure(CNKI), Wanfang Data Knowledge Service Platform(Wanfang), China Science and Technology Journal Database(VIP), Web of Science, PubMed, Embase, and Cochrane Library. The search period was from the creation of the databases to July 28, 2023. There were no restrictions on publication source or language. The searched MeSH terms are listed as follows: [“Transcranial Magnetic Stimulation” [MeSH] OR “Theta Burst Stimulation” OR “rTMS”] AND [“Stroke” [MeSH] OR “Cerebral Infarction” [MeSH] OR “Cerebral Hemorrhage” [MeSH] OR “Cerebrovascular Accident”] AND [“Cognitive Impairment” [MeSH] OR “Cognitive Function”]. In addition, a complementary search was conducted for references included in the literature. The search formula is available in Supplementary File.

### 2.4 Data extraction and management

The retrieved literature was imported into the EndNote software for centralized management. Two researchers (YW and LX) independently screened the literature based on the proposed inclusion and exclusion criteria. After excluding duplicate literature using EndNote, both researchers independently reviewed the titles, keywords, and abstracts of the articles to preliminarily exclude those that did not meet the inclusion criteria. Subsequently, they downloaded and thoroughly read the full text to determine whether it met the inclusion criteria. In case of any necessary clarifications or missing information, we contacted the original authors via email or phone. Data extraction was performed by researchers independently using a pre-designed data extraction form for further analysis. The extracted data included: first author, publication year, patient characteristics (gender, age, time to stroke onset), study-specific parameters (study type, number of enrolled patients, duration of treatment, interventions, outcome indicators, follow-up, adverse events), as well as application parameters of rTMS (frequency, number of pulses, intensity, stimulation site, and duration of each treatment). If there is any disagreement, it will be referred to a third researcher (LZ) to determine the final results.

### 2.5 Assessment of risk of bias

The included studies were assessed for bias using the risk assessment tools recommended by Cochrane (Cumpston et al., 2019), which encompassed randomization, assignment concealment, blinding procedures, blinding of outcome evaluation, completeness of outcome data, selective reporting of results, and identification of other potential biases. In cases where evaluators (YW and LX) held differing opinions, a third investigator (LZ) was consulted for evaluation. The risk of bias plots were generated using RevMan 5.3 software.

### 2.6 Data analysis

We analyzed data obtained from RCTs. A narrative synthesis was employed as a preferred method, given its suitability for summarizing studies with heterogeneous results (Trung et al., 2019). The findings were presented in tabular format using a narrative synthesis approach.

## 3 Results

### 3.1 Literature search results

**Figure 1:**
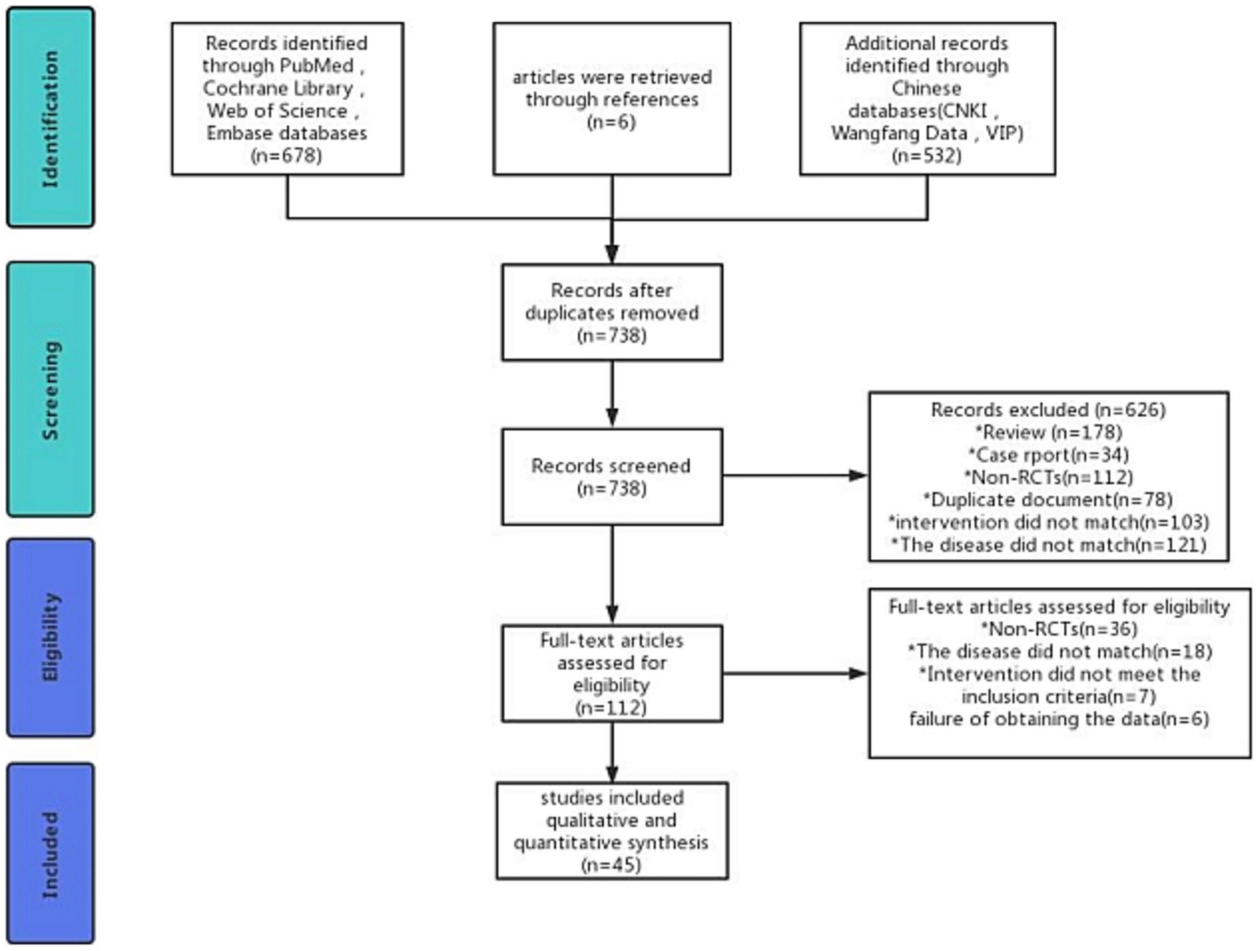
PRISMA flow diagram for study selection.

We searched 1,216 records and excluded 478 duplicates. After reading the title and abstract, 626 studies were excluded. After reading the complete text, we again excluded 67 studies. Eventually, 45 studies were considered for inclusion. The exclusion and screening process is detailed in Figure 1.

### 3.2 The characteristic of the included studies

45 RCTs were included, enrolling 3,066 patients with PSCI. Publication dates ranged from 2012 to 2023. RCTs included a minimum of 18 patients and a maximum of 200 patients. Adverse events were reported in 20 studies, as summarized in Table 1.

**Table 1:** Characteristics of the included studies.

Study	Sample Size (gender)		Age (y)		intervention		Time after onset (d)		Adverse events
	T (male/female)	C (male/female)	T	C	T	C	T	C	
Chen (2017)	15(11/4)	15 (9/6)	58.0 ± 8.9	60.1 ± 7.7	rTMS+ST	ST	35.7 ± 20.3	37.8 ± 16.1	No
Chen (2019)	70 (37/33)	70 (39/31)	57.1 ± 5.0	56.8 ± 4.8	rTMS+ST	ST	87 ± 24	2.8 ± 0.8	No
Ding (2019)	Group 1:15 (10/5); Group 2:14 (10/4); Group 3:15 (8/7)	14 (9/5)	Group 1:53.67 ± 7.58; Group 2:54.40 ± 6.82; Group 3:54.33 ± 7.72	53.53 ± 7.65	rTMS+ST	Sham rTMS+ST	Group 1:53.20 ± 18.06; Group 2:50.89 ± 17.64; Group 3:55.51 ± 15.54	49.49 ± 14.84	No
Duan (2016)	59 (35/24)	59 (36/23)	65.8 ± 7.1	66.4 ± 7.2	rTMS+ST	ST	132 ± 57	126 ± 48	NA
Gao (2021)	24 (11/13)	24 (14/10)	48.79 ± 6.61	48.58 ± 6.59	rTMS+ST	ST	41.72 ± 10.22	42.00 ± 9.66	NA
Gu (2012)	16 (8/8)	20 (12/8)	66.8 ± 8.1	71.7 ± 7.0	rTMS+ST	Sham rTMS+ST	NA	NA	No
Li (2020)	44 (26/18)	44 (24/20)	54.2 ± 6.3	56.4 ± 7.0	rTMS+ST	ST	NA	NA	NA
He (2017)	16 (9/7)	14 (8/6)	48.00 ± 12.42	47.43 ± 13.67	rTMS+ST	ST	46.8 ± 21.9	54.0 ± 23.1	NA
Jiang (2014)	30 (NA)	30 (NA)	60.41 ± 8.03	59.52 ± 7.19	rTMS+ST	ST	NA	NA	3 patients experienced headaches in the intervention group
Li (2023)	Group 1:50 (21/29); Group 2:50 (26/24); Group 3:50 (25/25)	50 (22/28)	Group 1:57.63 ± 5.23; Group 2:57.42 ± 5.22; Group 3:56.37 ± 5.12	56.91 ± 5.17	rTMS+ST	Sham rTMS+ST	Group 1:75.11 ± 15.05; Group 2:78.05 ± 15.61; Group 3:79.59 ± 15.89	78.68 ± 15.75	NA
Li (2022)	41 (24/17)	41 (23/18)	59.97 ± 4.71	60.18 ± 3.74	rTMS+ST	ST	29.87 ± 4.51	30.05 ± 4.16	In the intervention group, 4 patients had headache and 1 patient had nausea, while 5 patients in the control group had headache.
Li (2020)	50 (29/21)	50 (31/19)	58.94 ± 3.76	59.61 ± 3.75	rTMS+ST	ST	27.41 ± 4.23	28.74 ± 5.13	In the intervention group, 4 patients had headache, 1 patient had nausea and vomiting, while in the control group, 3 patients had headache, 2 patients had nausea and vomiting, 1 patient had diarrhea, and 1 patient had epilepsy.
Li (2015)	23 (11/12)	22 (12/10)	58.6 ± 4.2	57.9 ± 4.3	rTMS+ST	ST	120 ± 21	126 ± 24	2 patients in the intervention group complained of mild dizziness and headache discomfort
Ren (2018)	27 (14/13)	27 (13/14)	59.4 ± 7.1	58.6 ± 7.8	rTMS+ST	ST	50.0 ± 8.4	48.0 ± 10.7	NA
Li (2015)	32 (11/21)	30 (12/18)	58.2 ± 5.4	57.8 ± 6.1	rTMS+ST	ST	123 ± 24	129 ± 28	No
Liao (2017)	Group 1:30 (16/14); Group 2:30 (17/13)	30 (18/12)	Group 1:60.2 ± 6.5; Group 2:59.9 ± 6.7	60.5 ± 6.6	rTMS+ST	Sham rTMS+ST	Group 1:45.7 ± 5.3; Group 2:44.9 ± 5.7	45.8 ± 4.6	No
Lv (2020)	46 (28/18)	46 (29/17)	46.71 ± 10.98	46.35 ± 11.40	rTMS+ST	ST	123.0 ± 9.3	122.7 ± 8.7	Dizziness during rTMS treatment in 3 patients
Wu (2022)	Group 1:30 (17/13); Group 2:30 (18/12)	30 (16/14)	Group 1:55.4 ± 11.1; Group 2:54.2 ± 12.8	56.8 ± 11.7	Group 1:rTMS+AOT + ST; Group 2:rTms+ST	ST	Group 1:85.6 ± 31.3; Group 2:86.3 ± 29.7	88.7 ± 29.2	NA
Ma (2021)	37 (25/12)	38 (22/16)	60.95 ± 7.92	58.84 ± 10.89	rTMS+ST	Sham rTMS+ST	65.14 ± 26.62	63.63 ± 35.52	NA
Mao (2022)	Group 1:21 (16/5); Group 2:20 (14/6)	22 (16/6)	Group 1:58.48 ± 9.10; Group 2:60.00 ± 8.53	52.95 ± 14.62	rTMS+ST	Sham rTMS+ST	Group 1:62.10 ± 53.66; Group 2:46.86 ± 39.89	50.82 ± 30.90	NA
Pei (2022)	30 (18/13)	29 (22/7)	64.90 ± 5.46	66.93 ± 6.55	iTBS+ST	Sham iTBS+ST	90 ± 48	81 ± 48	NA
Tang (2015)	30 (17/13)	30 (18/12)	61.2 ± 10.8	60.9 ± 11.2	rTMS+ST	ST	NA	NA	No
Xu (2022)	65 (38/27)	65 (36/29)	64.09 ± 3.86	63.52 ± 4.27	rTMS+ST	ST	16.17 ± 3.54	15.64 ± 3.72	NA
Yin (2018)	12 (11/1)	13 (12/1)	58.58 ± 11.98	60.15 ± 10.29	rTMS+ST	Sham rTMS+ST	59.83 ± 30.59	56.15 ± 23.74	Transient headache during rTMS treatment in 3 patients
You (2023)	66 (39/27)	66 (35/31)	62.38 ± 4.44	62.13 ± 4.69	rTMS+ST	ST	NA	NA	NA
Yu (2019)	51 (29/22)	49 (25/24)	65.13 ± 10.32	62.15 ± 13.49	rTMS+ST	ST	397.5 ± 174.0	368.4 ± 193.5	NA
Zhang (2019)	30 (20/10)	30 (18/12)	58.44 ± 16.60	55.11 ± 18.03	rTMS+ST	ST	46.83 ± 28.13	49.00 ± 37.01	NA
Zhang (2022)	20 (11/9)	20 (8/12)	54 ± 7.0	57 ± 6	rTMS+ST	Sham rTMS+ST	180 ± 120	180 ± 120	NA
Zhang (2020)	Group 1:15 (8/7); Group 2:15 (7/8); Group 3:15 (9/6); Group 4:15 (8/7)	15 (10/5)	Group 1:59.87 ± 6.24; Group 2:59.47 ± 6.68; Group 3:55.20 ± 8.07; Group 4:58.00 ± 5.84	56.80 ± 9.69	rTMS+ST	Sham rTMS+ST	Group 1:147.0 ± 60.9; Group 2:143.1 ± 43.2; Group 3:153.9 ± 59.7; Group 4:150.9 ± 58.2	147.0 ± 61.8	2 patients developed dizziness after 10 Hz rTMS treatment
Zhang (2021)	21 (15/6)	22 (14/8)	60.67 ± 9.53	58.98 ± 7.88	rTMS+ST	Sham rTMS+ST	51.90 ± 21.90	49.59 ± 29.39	NA
Zhou (2017)	15 (9/6)	15 (11/4)	55.14 ± 10.76	53.41 ± 11.29	rTMS+ST	ST	124.5 ± 68.4	128.1 ± 114.6	NA
Zheng (2020)	55 (36/19)	51 (33/18)	58.3 ± 7.9	59.7 ± 6.3	rTMS+ST	Sham rTMS+ST	48.7 ± 14.4	47.3 ± 11.8	One patient in the intervention group had poor concentration, one patient complained of sleep disturbance, and one patient in the control group had dizziness
Zuo (2013)	53 (30/23)	49 (27/22)	62.6 ± 7.3	64.3 ± 7.8	rTMS+ST	ST	NA	NA	NA
Bi (2022)	18 (13/5)	18 (12/6)	60.39 ± 10.87	59.50 ± 11.25	rTMS+ST	Sham rTMS+ST	58.11 ± 28.89	58.39 ± 24.70	No
Kim (2010)	Group 1:6 (2/4); Group 2:6 (4/2)	6 (4/2)	Group 1:68.3 ± 7.4; Group 2:53.5 ± 16.9	66.8 ± 17.2	rTMS+ST	Sham rTMS+ST	Group 1:404 ± 71.7; Group 2:241.2 ± 42.59	69.7 ± 39	NA
Li (2021)	33 (21/12)	32 (19/13)	61.79 ± 5.51	59.47 ± 6.75	rTMS+ST	Sham rTMS+ST	28.64 ± 12.60	27.78 ± 11.01	No
Li (2022)	28 (16/12)	30 (18/12)	69.14 ± 14.06	64.57 ± 17.12	iTBS+ST	Sham iTBS+ST	23.93 ± 10.16	24.29 ± 9.34	NA
Lu (2015)	19 (12/7)	21 (13/8)	42.5 ± 12.3	47.3 ± 11.8	rTMS+ST	Sham rTMS+ST	106.85 ± 90.75	86.99 ± 70.39	NA
Tsai (2020)	Group 1:15 (11/4); Group 2:11 (9/2)	15 (13/2)	Group 1:60.13 ± 14.1; Group 2:57.45 ± 12.3	56.23 ± 12	Group 1:rTMS+ST; Group 2:iTBS+ST	Sham rTMS+ST	Group 1:554.1 ± 606.3; Group 2:998.1 ± 792.0	1,140 ± 237	One patient in the intervention group developed transient headache and one patient developed dizziness, while one patient in the control group developed headache.
Li (2020)	15 (7/8)	15 (9/6)	65.47 ± 3.68	64.53 ± 4.72	rTMS+ST	Sham rTMS+ST	22.73 ± 8.05	19.13 ± 7.95	NA
Park (2015)	10 (4/6)	10 (5/5)	NA	NA	CACR+ST	rTMS+ST	NA	NA	NA
Liu (2020)	29 (10/19)	29 (16/13)	58.55 ± 6.24	57.69 ± 7.25	rTMS+ST	Sham rTMS+ST	263.7 ± 55.2	258.6 ± 55.2	NA
Wang (2021)	Group 1:15 (10/5); Group 2:15 (11/4)	15 (10/5)	Group 1:60.2 ± 11.6; Group 2:57.8 ± 13.0	58.8 ± 9.3	rTMS+ST	Sham rTMS+ST	Group 1:56.0 ± 25.4; Group 2:52.5 ± 21.6	57.3 ± 18.5	No
Qi (2021)	36 (NA)	36 (NA)	59.6 ± 8.0	58.7 ± 7.9	rTMS+ST	ST	1.45 ± 0.21	1.45 ± 0.20	NA
Hu (2016)	30 (14/16)	30 (15/15)	57.5 ± 13.3	56.2 ± 10.9	rTMS+ST	ST	NA	NA	2 patients in the intervention group developed headache and dizziness

T, treatment group; C, control group; y, year; m, month; d, day; rTMS, repetitive transcranial magnetic stimulation; ST, standard rehabilitation treatment; CACR, computer-assisted cognitive rehabilitation; iTBS, intermittent theta burst stimulation; AOT, action observation therapy; NA, not available.

### 3.3 Results of bias risk assessment

**Figure 2:**
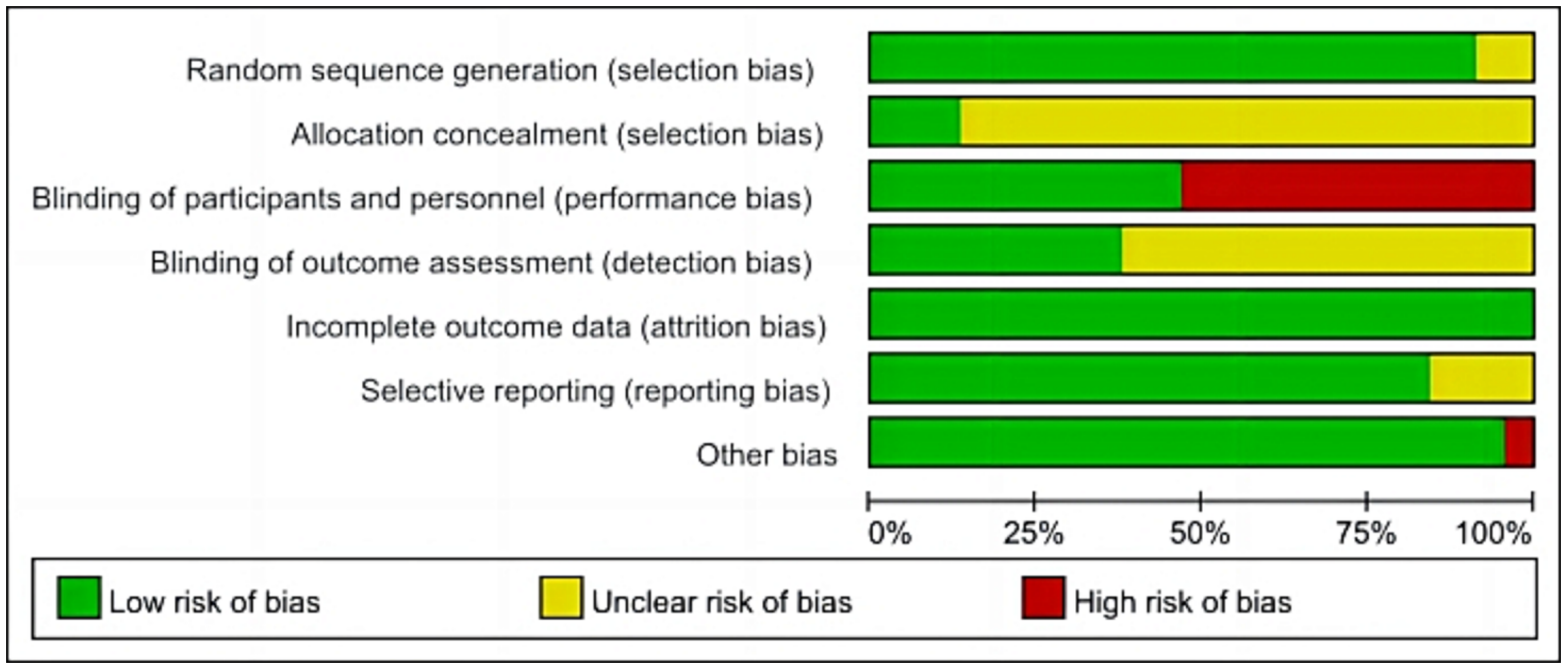
Risk of bias graph.

**Figure 3:**
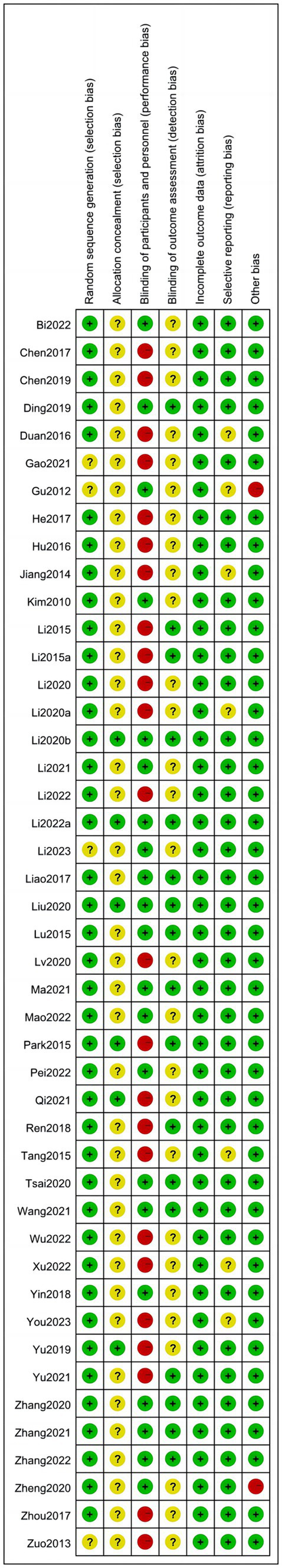
Risk of bias summary.

We assessed the risk of bias in the included RCTs. 24 RCTs (Zuo and Zhang, 2013; Jiang et al., 2014; Li et al., 2015a,b; Park and Yoon, 2015; Tang et al., 2015; Duan and Ding, 2016; Hu et al., 2016; Chen et al., 2017; He and Zhou, 2017; Zhou et al., 2017; Ren et al., 2018; Chen et al., 2019; Yu and Zhang, 2019; Zhang and Zhou, 2019; Li et al., 2020a,b; Lv et al., 2020; Gao et al., 2021; Qi et al., 2021; Li et al., 2022; Wu et al., 2022; Xu et al., 2022; You et al., 2023) did not use sham rTMS in the control group, which may not have blinded patients and implementers, resulting in a high risk of bias. In addition, only seven studies (Park and Yoon, 2015; Yu and Zhang, 2019; Li et al., 2020c; Liu et al., 2020; Qi et al., 2021; Li et al., 2022) reported on allocation concealment, and 16 (Li et al., 2015a,b; Lu et al., 2015; Park and Yoon, 2015; Liao et al., 2017; Ren et al., 2018; Ding et al., 2019; Zhang and Zhou, 2019; Li et al., 2020c; Liu et al., 2020; Tsai et al., 2020; Zhang et al., 2020; Ma et al., 2021; Zhang et al., 2021; Li et al., 2022; Zhang et al., 2022) reported on assessor blindness. The specific results are presented in Figures 2–3.

### 3.4 rTMS application parameters

The application characteristics of rTMS were shown in Table 2.

**Table 2:** Repetitive transcranial magnetic stimulation intervention parameters.

Authors	Intensity	Frequency	Stimulus area	Total pulses	Time of each treatment	Number of sessions (Frequency)	Course of treatment
Chen (2017)	90% ~ 120% RMT	0.5 ~ 1.0 Hz (Healthy side)/5 ~ 10 Hz (Diseased side)	DLPFC (Diseased/Healthy side)	900-1200 (Healthy side)/600-800 (Diseased side)	NA	20 (5 days/weeks)	4 weeks
Chen (2019)	80% RMT	10 Hz	DLPFC (Left side)	NA	20 min	20 (5 days/weeks)	4 weeks
Ding (2019)	110% RMT	Group 1:5 Hz; Group 2:1 Hz; Group 3:5 Hz/1 Hz	Group 1:DLPFC (Diseased side); Group 2:DLPFC (Healthy side); Group 3:DLPFC (Diseased/Healthy side)	NA	20 min	12 (6 days/weeks)	2 weeks
Duan (2016)	80% RMT	3 Hz	Bilateral frontal, temporal and occipital lobes	600	NA	NA	2 weeks
Gao (2021)	120% RMT	10 Hz	DLPFC (Diseased side)	NA	20 min	24 (6 days/weeks)	4 weeks
Gu (2012)	110% RMT	5 Hz	DLPFC (Left side)	6,000	NA	10 consecutive days	10 days
Li (2020)	70% RMT	1 Hz	NA	600	20 min	20 (5 days/weeks)	4 weeks
He (2017)	80 ~ 120% RMT	10 Hz	DLPFC (Diseased side)	NA	20 min	20 (5 days/weeks)	4 weeks
Jiang (2014)	120% RMT	10 Hz	DLPFC (Left side)	3,000	NA	25 (5 days/weeks)	5 weeks
Li (2023)	80% RMT	Group 1:1 Hz; Group 2:10 Hz; Group 3:10 Hz/1 Hz	Group 1:DLPFC (Healthy side); Group 2:DLPFC (Diseased side); Group 3:DLPFC (Diseased/Healthy side)	NA	20 min	10 (5 days/weeks)	2 weeks
Li (2022)	80% RMT	10 Hz	NA	NA	20 min	20 (5 days/weeks)	4 weeks
Li (2020)	100% RMT	0.5 Hz	DLPFC (Diseased side)	NA	20 min	20 (5 days/weeks)	4 weeks
Li (2015)	80% RMT	5 Hz	DLPFC (Left side)	6,000	20 min	20 (5 days/weeks)	4 weeks
Ren (2018)	80% RMT	3 Hz	DLPFC (Left side)	6,000	20 min	20 (5 days/weeks)	4 weeks
Li (2015)	80% RMT	5 Hz	DLPFC (Left side)	NA	20 min	20 (5 days/weeks)	4 weeks
Liao (2017)	80% RMT	Group 1:0.5 Hz; Group 2:3 Hz	DLPFC (Left side)	NA	20 min	20 (5 days/weeks)	4 weeks
Lv (2020)	80% RMT	10 Hz	DLPFC (Left side)	NA	20 min	20 (5 days/weeks)	4 weeks
Wu (2022)	80% RMT	1 Hz/5 Hz	DLPFC (Healthy side)	NA	20 min (1 Hz)/10 min (5 Hz)	20 (5 days/weeks)	4 weeks
Ma (2021)	90% RMT	1 Hz	DLPFC (Healthy side)	1,000	20 min	20 (5 days/weeks)	4 weeks
Mao (2022)	80% RMT	Group 1:10 Hz; Group 2:1 Hz	Group 1:DLPFC (Left side); Group 2:DLPFC (Right side)	Group 1:2000;Group 2:1600	20 min	18 (6 days/weeks)	3 weeks
Pei (2022)	80% RMT	3 pulses of 50 Hz in 1 group, each group stimulated repeatedly at 5 Hz (2 s on, 8 s off)	DLPFC (Left side)	1,200	383.68 s	20 (5 days/weeks)	4 weeks
Tang (2015)	110% RMT	5 Hz	DLPFC (Left side)	600	NA	24 (5 days/weeks)	4 weeks
Xu (2022)	80% RMT	20 Hz	DLPFC (bilateral)	NA	30 min	40 (5 days/weeks)	8 weeks
Yin (2018)	80% RMT	10 Hz	DLPFC (Left side)	2000	20 min	20 (5 days/weeks)	4 weeks
You (2023)	NA	10 Hz	DLPFC (Left side)	NA	20 min	40 (5 days/weeks)	8 weeks
Yu (2019)	80% RMT	10 Hz	DLPFC (Left side)	NA	20 min	40 (5 days/weeks)	8 weeks
Zhang (2019)	80% RMT	5 Hz	DLPFC (Left side)	1,600	20 min	20 (5 days/weeks)	4 weeks
Zhang (2022)	80% RMT	5 Hz	DLPFC (Left side)	3,000	20 min	20 (5 days/weeks)	4 weeks
Zhang (2020)	Group 1/2:90% MT; Group 3/4:100% MT	Group 1:0.5 Hz; Group 2:1 Hz; Group 3:5 Hz; Group 4:10 Hz	Group 1/2:DLPFC (Right side); Group 3/4:DLPFC (Right side)	Group 1/2:600; Group 3/4:6000	20 min	20 (5 days/weeks)	4 weeks
Zhang (2021)	90% RMT	1 Hz	DLPFC (Healthy side)	1,000	20 min	20 (5 days/weeks)	4 weeks
Zhou (2017)	80% RMT	1 Hz	DLPFC (Healthy side)	NA	20 min	40 (5 days/weeks)	8 weeks
Zheng (2020)	80% RMT	10 Hz	DLPFC (Left side)	NA	20 min	20 (5 days/weeks)	4 weeks
Zuo (2013)	80% RMT	5 Hz	Bilateral frontal, temporal and occipital lobes	600	NA	20 (5 days/weeks)	4 weeks
Bi (2022)	80% RMT	1 Hz	DLPFC (Healthy side)	600	NA	40 (5 days/weeks)	8 weeks
Kim (2010)	80% RMT	Group 1:10 Hz; Group 2:1 Hz	DLPFC (Left side)	Group 1:450; Group 2:900	Group 1:30 min; Group 2:20 min	10 (5 days/weeks)	2 weeks
Li (2021)	90% RMT	1 Hz	DLPFC (Healthy side)	1,000	20 min	20 (5 days/weeks)	4 weeks
Li (2022)	NA	3 pulses of 50 Hz in 1 group, each group stimulated repeatedly at 5 Hz (2 s on, 8 s off)	DLPFC (Left side)	600	192 s	10 (5 days/weeks)	2 weeks
Lu (2015)	100% RMT	1 Hz	DLPFC (Right side)	600	NA	20 (5 days/weeks)	4 weeks
Tsai (2020)	80% RMT	Group 1:3 pulses of 50 Hz in 1 group, each group stimulated repeatedly at 5 Hz (2 s on, 8 s off); Group 2:5 Hz	DLPFC (Left side)	Group 1:600; Group 2:600	Group 1:190 s; Group 2:10 min	10 (5 days/weeks)	2 weeks
Li (2020)	100% RMT	5 Hz	DLPFC (Left side)	2000	NA	15 (5 days/weeks)	3 weeks
Park (2015)	100% RMT	10 Hz	DLPFC (Healthy side)	1,000	20 min	12 (3 days/weeks)	4 weeks
Liu (2020)	90% RMT	10 Hz	DLPFC (Left side)	700	NA	20 (5 days/weeks)	4 weeks
Wang (2021)	80% RMT	Group 1:10 Hz; Group 2:1 Hz	Group 1:DLPFC (Diseased side); Group 2:DLPFC (Healthy side)	NA	20 min	40 (5 days/weeks)	8 weeks
Qi (2021)	80% RMT	3 Hz	DLPFC (Left side)	6,000	20 min	12 (3 days/weeks)	4 weeks
Hu (2016)	100% RMT	10 Hz	DLPFC (Diseased side)	3,000	NA	20 (5 days/weeks)	4 weeks

RMT, resting motor potential threshold; DLPFC, dorsolateral prefrontal cortex; min, minute; s, second.

#### 3.4.1 Stimulation frequency

There was no standardized protocol for the application frequency of rTMS in the included studies. Based on frequency, rTMS could be categorized into high-frequency repetitive transcranial magnetic stimulation (HF-rTMS) and low-frequency repetitive transcranial magnetic stimulation (LF-rTMS). Among low-frequency protocols, 1 Hz was the most commonly utilized, followed by 0.5 Hz. For high-frequency applications, 10 Hz was predominantly employed, although researchers had also explored rTMS frequencies of 3 Hz, 5 Hz, and 20 Hz. In all instances, rTMS consistently demonstrated significant improvements in cognitive function among patients. Based on the event-related potential (ERP) test results, Wu (Wu et al., 2022) applied stimulation to the bilateral dorsolateral prefrontal cortex (DLPFC), temporal lobe, precentral gyrus, and posterior 1/3 of inferior frontal gyrus. The selection of frequency was guided by the excitability of the target area: 1 Hz low-frequency rTMS was chosen for areas with higher excitability, while 5 Hz high-frequency rTMS was selected for areas with lower excitability.

However, it was noteworthy that seven studies (Kim et al., 2010; Liao et al., 2017; Ding et al., 2019; Zhang et al., 2020; Wang et al., 2021b; Mao et al., 2022; Li et al., 2023) had assessed the clinical effects of rTMS at various frequencies, and most of them had reported that both LF-rTMS and HF-rTMS could improve cognitive function (MoCA (Liao et al., 2017; Ding et al., 2019; Zhang et al., 2020; Wang et al., 2021b; Li et al., 2023), LOTCA (Wang et al., 2021b), MMSE (Zhang et al., 2020; Li et al., 2023), TOLT (Kim et al., 2010)), balance (Li et al., 2023), daily living ability (Kim et al., 2010; Liao et al., 2017; Li et al., 2023) and prolonged auditory event-related potential P3000 (AERP P3000) latency and promoted wave amplitude (Liao et al., 2017; Ding et al., 2019; Zhang et al., 2020). Interestingly, no significant difference in efficacy between these two frequencies had been observed. Only Mao (Mao et al., 2022), in a comparison of efficacy between 1 Hz and 10 Hz, found that at 10 Hz, rTMS was able to achieve more positive effects in the results of MMSE, Fuel-Meyer assessment (FMA), Modified Barthel Index (MBI), and MoCA. Conversely, the combination of high-frequency and low-frequency stimulation had been shown to yield superior clinical outcomes (Chen et al., 2017; Ding et al., 2019; Li et al., 2023). Ding (Ding et al., 2019) demonstrated that the 5 Hz group, 1 Hz group, and 5 Hz combined with the 1 Hz group (with high frequency applied to the affected side DLPFC and low frequency applied to the healthy side DLPFC, alternating stimulus) exhibited improvements in MOCA score, reduced AERP P300 latency and increased the amplitude. Notably, the combined group displayed the most significant effects. At the 8-week follow-up assessment, all rTMS treatment groups showed improvement compared to baseline measures as well as outperformed the control group. Furthermore, the combined group remained significantly superior to both the 5 Hz group and the 1 Hz group (*p* < 0.05).

#### 3.4.2 Stimulus intensity

Regarding the application intensity, all studies were conducted based on the patient’s resting motor potential threshold (RMT). The majority of studies utilized an intensity of 80% RMT, while some employed intensities of 70% RMT, 90% RMT, 100% RMT, 110% RMT, and 120% RMT. Notably, all stimulation intensities yielded positive outcomes. In terms of RMT detection methodology, most studies opted to measure the first interosseous dorsal muscle or abductor pollicis brevis. The minimum magnetic stimulation intensity capable of eliciting a muscle motor evoked potential (amplitude ≥50 μV) in at least five out of ten consecutive stimulations was considered as the RMT value.

#### 3.4.3 Stimulus area

As for the site of rTMS, the dorsolateral prefrontal cortex (DLPFC) had been predominantly utilized in most studies, although some (Zuo and Zhang, 2013; Duan and Ding, 2016) had opted for bilateral frontal lobe, temporal lobe, and occipital lobe as stimulation sites. However, the selection of DLPFC location for rTMS also varied depending on the stroke site of patients. Certain studies (Park and Yoon, 2015; Hu et al., 2016; Chen et al., 2017; He and Zhou, 2017; Zhou et al., 2017; Ding et al., 2019; Li et al., 2020a; Gao et al., 2021; Li et al., 2021; Ma et al., 2021; Wang et al., 2021b; Zhang et al., 2021; Wu et al., 2022; Yingli et al., 2022; Li et al., 2023) had employed the affected side DLPFC as the high-frequency stimulation area while designating the healthy side DLPFC as the low-frequency stimulation area. Furthermore, certain studies (Kim et al., 2010; Gu et al., 2012; Jiang et al., 2014; Li et al., 2015a,b; Tang et al., 2015; Liao et al., 2017; Ren et al., 2018; Yin et al., 2018; Chen et al., 2019; Yu and Zhang, 2019; Zhang and Zhou, 2019; Li et al., 2020c; Liu et al., 2020; Lv et al., 2020; Tsai et al., 2020; Zhang et al., 2020; Zheng et al., 2020; Qi et al., 2021; Li et al., 2022; Mao et al., 2022; Pei et al., 2022; Zhang et al., 2022; You et al., 2023) solely targeted one side of DLPFC without considering patient-specific stroke locations.

#### 3.4.4 Stimulation pulse count

The parameter of pulse count is crucial in rTMS research. Specifically, the range of pulse counts includes 450, 600, 700, 900, 1,000, 1,200, 1,500, 1,600, 2000, 3,000, and even up to a maximum of 6,000 pulses. Notably, Clinical trial evidence consistently demonstrates the efficacy of rTMS in enhancing cognitive function among patients with PSCI.

#### 3.4.5 Duration of each treatment session

Each session lasted between 10 and 30 min, with the most prevalent treatment duration being 20 min. Kim et al. (2010) employed a 20-min treatment duration for low-frequency stimulation at 1 Hz and extended it to a 30-min duration for high-frequency stimulation at 10 Hz. In all instances, the outcomes exhibited significant positive effects.

#### 3.4.6 Duration and frequency of treatment

The duration of treatment also exhibits variations, ranging from a minimum of 10 days to a maximum of 8 weeks. Most rTMS interventions typically span over 4 weeks. However, certain studies opt for intervention periods of either two or 3 weeks. Both high-frequency and low-frequency rTMS protocols commonly employ 2 weeks. Furthermore, the frequency of interventions varies between three times per week and up to six times per week, with the most prevalent being an intervention schedule consisting of five sessions per week.

### 3.5 iTBS application parameters

Three RCTs (Tsai et al., 2020; Li et al., 2022; Pei et al., 2022) were conducted to investigate the efficacy of intermittent theta burst stimulation (iTBS) in treating patients with PSCI. The left DLPFC was consistently chosen as the targeted stimulation site across all three studies. In two studies (Tsai et al., 2020; Pei et al., 2022), a stimulation intensity of 80% RMT was utilized, while Li et al. (2022) did not specify the exact stimulation intensity employed. The standardized stimulation pattern consisted of three consecutive pulses at 50 Hz, repeated at a frequency of 5 Hz (2 s on, 8 s off). Both Li et al. (2022) and Tsai et al. (2020) adopted a total pulse count of 600 over 192 s, whereas Pei et al. (2022) opted for a longer protocol with 1,200 pulses lasting for approximately 383.68 s. Regarding the frequency of intervention sessions, all three studies implemented once-daily sessions for 5 days per week. Li et al. (2022) and Tsai et al. (2020) conducted the treatment for 2 weeks, while Pei et al. (2022) administered a four-week course. Positive therapeutic effects were observed across all included studies.

### 3.6 rTMS combined with other treatments

Most studies had combined rTMS with various physical therapy techniques, encompassing neurodevelopmental therapy, motor relearning programs, activities of daily living training, guessing games, visual tracking, picture memory, recall picture sequence, short passage recitation, building blocks, chess playing, puzzles, number identification, looking for differences, maze games, making handicrafts. Additionally, interventions such as acupuncture, hyperbaric oxygen therapy, action observation therapy (AOT), Forbrain speech and auditory feedback training, computer-assisted cognitive rehabilitation (CACR), as well as conventional drug therapies including platelet inhibitors, lipid-lowering agents antihypertensive drugs hypoglycemic drugs, cholinesterase inhibitors, N-methyl-d-aspartic acid receptor antagonists, free radical scavengers, microcirculation promoters had been included in the studies. Positive effects were observed in both intervention groups and control groups with even better results achieved through combined use.

It was noteworthy that certain studies employed a combination of CACR and rTMS. CACR referred to a computer-based cognitive training program encompassing exercises and games, utilizing multimedia, informatics resources, as well as specific hardware and software systems to implement comprehensive cognitive training in memory, attention, problem-solving, job simulation, language proficiency, practice repetition, and processing speed. The combined treatment group exhibited significant improvements in patients’ cognitive function (Lu et al., 2015; Park and Yoon, 2015; Yin et al., 2018; Zhang and Zhou, 2019; Zheng et al., 2020; Pei et al., 2022). Additionally, Park and Yoon (2015) observed that compared to 10 Hz rTMS treatment alone, CACR demonstrated more pronounced enhancements in Lowenstein Occupational Therapy Cognitive Assessment-Geriatric (LOTCA-G).

### 3.7 Safety

We conducted a safety analysis of the included RCTs. Adverse events were reported in 20 RCTs (Gu et al., 2012; Jiang et al., 2014; Li et al., 2015a,b; Tang et al., 2015; Hu et al., 2016; Chen et al., 2017; Liao et al., 2017; Yin et al., 2018; Chen et al., 2019; Ding et al., 2019; Li et al., 2020a; Lv et al., 2020; Tsai et al., 2020; Zhang et al., 2020; Zheng et al., 2020; Li et al., 2021; Wang et al., 2021b; Li et al., 2022; Yingli et al., 2022), while no adverse events were reported in 10 studies (Gu et al., 2012; Li et al., 2015b; Tang et al., 2015; Chen et al., 2017; Liao et al., 2017; Chen et al., 2019; Ding et al., 2019; Li et al., 2021; Wang et al., 2021b; Yingli et al., 2022). Conversely, adverse events following rTMS treatment encompassed headache (Jiang et al., 2014; Li et al., 2015a; Hu et al., 2016; Yin et al., 2018; Li et al., 2020a; Tsai et al., 2020; Li et al., 2022), dizziness (Li et al., 2015a; Hu et al., 2016; Lv et al., 2020; Tsai et al., 2020; Zhang et al., 2020), nausea and vomiting (Li et al., 2020a, 2022), sleep disturbances (Zheng et al., 2020), and poor concentration (Zheng et al., 2020). After rest, patients experienced relief from their symptoms. No studies had reported the occurrence of epilepsy, cerebral hemorrhage, or secondary cerebral infarction resulting from rTMS. Li et al. (2015a) observed no alterations in blood analysis results, urine analysis results, fecal analysis results, liver function, renal function, blood lipid spectrum, myocardial enzyme levels, electrocardiogram readings, or other tests before and after treatment.

### 3.8 Publication bias

We employed a funnel plot analysis to assess the presence of publication bias in MOCA, MMSE, AERP P3000 latency, and AERP P3000 amplitude. The findings revealed that the distribution of MOCA and AERP P3000 latency exhibited relative symmetry, suggesting a potential absence of publication bias. Conversely, the distribution of MMSE and AERP P3000 amplitude displayed noticeable asymmetry, indicating the presence of publication bias, as shown in Supplementary file.

## 4 Discussion

After conducting a comprehensive analysis of the included RCTs, we have observed that rTMS exhibits significant efficacy in enhancing cognitive function and improving the quality of life among patients with PSCI. The clinical effects of rTMS vary depending on different treatment parameters. Notably, combining rTMS with pharmacotherapy or cognitive rehabilitation training has demonstrated superior therapeutic effects.

There were variations in the selection of rTMS frequencies across different RCTs. In clinical practice, LF-rTMS commonly employs parameters of 0.5 Hz and 1 Hz, while HF-rTMS includes frequencies of 3 Hz, 5 Hz, 10 Hz, and 20 Hz. rTMS at different frequencies can modulate the excitability of the stimulated site or cerebral cortex by either inhibiting or facilitating neuronal activity. For instance, LF-rTMS (≤1 Hz) can suppress local neuronal activity and reduce cerebral cortex excitability. Conversely, HF-rTMS (>1 Hz) can enhance local neuronal function and increase cerebral cortex excitability (Lefaucheur et al., 2020). LF-rTMS can modulate the plasticity of hippocampal neuronal synapses through the BDNF–TrkB pathway, thereby enhancing learning and memory abilities (Ma et al., 2013). Additionally, it can reduce triiodothyronine levels to decrease oxygen consumption and metabolic rate in the body, facilitating the repair of injured sites (Alevizaki et al., 2007). Studies (Devanand et al., 2010) have revealed that cognitive dysfunction after stroke is primarily caused by decreased blood flow in the lesion itself and brain tissues such as the “ischemic penumbra.” However, high-frequency rTMS has been shown to increase average blood flow in the middle cerebral artery (Ogiue-Ikeda et al., 2005; Duering et al., 2011), improve brain cell glucose metabolism, mitigate secondary cerebral ischemia damage, and enhance cognitive function (Kozel et al., 2011). Furthermore, high-frequency rTMS can improve tolerance of functional damage caused by ischemia in hippocampal and other nerve tissues. It promotes the survival of hippocampal neurons while inhibiting cell apoptosis. Moreover, it regulates excitability in both local and remote cerebral cortexes to achieve cortical functional area reconstruction. This process also facilitates the growth and repair of white matter in brain-injured areas (Kozel et al., 2011; Zhao et al., 2012). It is important to emphasize that in the comparison of the efficacy of HF-rTMS and LF-rTMS on cognitive function, most RCTs (Kim et al., 2010; Liao et al., 2017; Ding et al., 2019; Zhang et al., 2020; Wang et al., 2021b; Li et al., 2023) did not demonstrate significant differences between the two interventions. This finding aligns with previous meta-analyses (Gong et al., 2023; Yin et al., 2023). Conversely, combining HF-rTMS and LF-rTMS has shown more favorable outcomes (Chen et al., 2017; Ding et al., 2019; Li et al., 2023). Furthermore, studies (Boggio et al., 2010; Kim et al., 2010) have indicated that HF-rTMS yields superior effects on psychological emotions compared to LF-rTMS.

The efficacy of rTMS is also influenced by the choice of stimulation site, with the DLPFC being commonly selected in most RCTs. Several studies (Sun et al., 2012; Van den Boom et al., 2018) have suggested that cognitive brain regions are predominantly situated within the bilateral DLPFC, which serves as a crucial hub in the brain network and exhibits close associations with cognitive functions such as memory, reasoning, and attention. Based on the dominant hemisphere theory (there is a structural and functional asymmetry between the right and left hemispheres of the brain, the left hemisphere of a right-handed person is mostly the dominant hemisphere, which is mainly responsible for speech, computation, writing, and logical reasoning.) and the human brain connectivity group theory (the brain is made up of multiple neurons, neuron clusters, or multiple brain regions that are connected to form a heterogeneous network structure, which accomplishes a wide range of brain functions through interactions) (Zheng et al., 2020). Since the RCTs chose to include right-handed patients for observation, the left DLPFC was mainly chosen as the stimulation site in the clinic. In addition, some studies have chosen the affected side of DLPEF as the HF-rTMS target area, while selecting the healthy side of DLPEF as the LF-rTMS target area. This selection may be attributed to the fact that under normal circumstances, there exists a balanced state of mutual inhibition between the left and right cerebral hemispheres in humans. However, brain damage disrupts this balance between hemispheres. Following a stroke, reduced inhibition from the damaged hemisphere on the unaffected hemisphere is likely to result in increased excitability of the unaffected hemisphere and heightened inhibition of the damaged hemisphere (Seniów et al., 2013). Consequently, clinical researchers aim to enhance cortical excitability by applying HF-rTMS to stimulate the affected side of DLPEF and reduce cortical excitability by employing LF-rTMS on the healthy side of DLPEF. This approach helps facilitate neural circuitry reorganization and normalize activation patterns across both hemispheres. It is plausible that this combined LF-rTMS and HF-rTMS strategy contributes to achieving superior outcomes.

Stimulus intensity exhibited variability, ranging from 70% RMT to 120% RMT, with the majority of RCTs opting for 80% RMT. Positive outcomes were observed across all stimulus intensities in rTMS. Furthermore, varying numbers of pulse stimulations also demonstrated improvements in cognitive function among patients, despite Kim et al. (2010) employing only 450 pulses. This observation may be attributed to both the stimulation intensity and number of pulse stimulations surpassing the threshold required for modulating cerebral cortex excitability and brain networks. It was found that patients with PSCI who underwent rTMS treatment at a frequency of 5 Hz and intensity set at 100% RMT exhibited significantly increased fractional amplitude of low-frequency fluctuation in specific brain regions, including the superior temporal gyrus, inferior frontal gyrus, and parahippocampal gyrus. Furthermore, there was an observed enhancement in functional connectivity between the LDPFC and precuneus, middle frontal gyrus, inferior frontal gyrus, inferior temporal gyrus, as well as limbic gyrus (Li et al., 2020c). Another study conducted rTMS treatment on 86 PSCI patients using a frequency of 5 Hz and intensity set at 80% RMT. Compared to pre-treatment values, significant decreases were found in the regional homogeneity values of the left superior frontal gyrus, right lobe, right superior marginal gyrus, left middle frontal gyrus, right inferior frontal gyrus, and other regions. Conversely, significant increases were observed in the regional homogeneity values of the left middle temporal gyrus, right inferior temporal gyrus, left superior temporal gyrus, right cerebellar hemisphere, and other regions. Additionally, a decrease in amplitude of low-frequency fluctuation (ALFF) was noted in the right anterior central gyrus while an increase was seen in ALFF within the right cerebellar Crus2 region and left inferior parietal angular gyri region (Li et al., 2023). The aforementioned findings demonstrate that rTMS can modulate neural excitability within specific cerebral regions while enhancing similarity and functional connectivity across distinct brain areas to facilitate recovery of cognitive function. However, it is important to note that high-intensity stimulation or excessive pulsed stimulation may elevate the risk for epilepsy development. Moreover, a higher incidence of dizziness and headache adverse events had been observed in RCTs encompassing a substantial number of pulses (Jiang et al., 2014; Li et al., 2015a; Hu et al., 2016; Yin et al., 2018; Zhang et al., 2020).

During each treatment session, the duration ranged from 20 to 30 min. However, it is important to note that longer treatment durations did not yield more satisfactory outcomes. In most cases of single-frequency rTMS interventions, a total intervention duration of 4 weeks was commonly employed, with a minimum duration of 10 days and a maximum duration of 8 weeks. Conversely, combination treatments involving HF-rTMS and LF-rTMS opted for either two or 4 weeks as the overall intervention period.

There were variations in the frequency of interventions, ranging from three to seven treatments per week (with only one treatment administered per day), with the majority of studies opting for five treatments per week. The rTMS stimulation has a cumulative effect, resulting in persistent biological effects even after the end of stimulation. Furthermore, each rTMS stimulus can be stored as a “memory” in the stimulated area, leading to new effects when subsequent stimuli are applied (Valero-Cabre et al., 2008). Repetitive stimulation activates subcortical neural network structures and modifies synaptic plasticity, thereby enhancing cognitive function (Rossi et al., 2009; Selimbeyoglu and Parvizi, 2010). However, there may exist an upper threshold for this cumulative effect. Prolonged and high-intensity application of high-frequency stimulation fails to yield superior outcomes and may even result in adverse consequences such as epilepsy, syncope, headache, cognitive or neuropsychological changes, and acute mental alterations (Rossi et al., 2009; Levkovitz et al., 2015).

iTBS represents an optimized form of rTMS, characterized by the advantages of employing low stimulus intensity, short stimulus cycles, and long-term benefits (Nowak et al., 2010). It exerts its effects on regulating neuroplasticity and excitability in the brain by reducing inhibitory control mechanisms and inducing neurotransmitter release (Hoy et al., 2016; Trung et al., 2019; Gebreegziabhere et al., 2022). Although studies (Tsai et al., 2020; Li et al., 2022; Pei et al., 2022) have demonstrated the efficacy of iTBS in improving cognitive function and enhancing daily activities in patients with PSCI, further exploration is required to establish whether iTBS surpasses traditional rTMS, given the limited clinical evidence available from evidence-based medicine. Notably, Tsai et al. (2020) findings not only failed to identify any differences in efficacy between iTBS and 5 Hz rTMS but also indicated superior attention regulation effects within the 5 Hz rTMS group.

### 4.1 Strengths and limitations

It is worth emphasizing that this study included a total of 45 RCTs. We conducted an in-depth analysis of the variability in rTMS efficacy across different parameters and further explored its potential mechanisms of action. These findings provide evidence-based medical support for the clinical utilization of rTMS. Naturally, this study does have certain limitations. Firstly, the sample size of some included RCTs was relatively small, potentially impacting the reliability of the findings. Additionally, given that a majority of the studies originate from China, there may be a regional bias present. Secondly, as the clinical efficacy stems from multiple parameters working in conjunction with each other, it is worth noting that this study solely focuses on analyzing the impact of individual parameters within RCTs and lacks an assessment of their combined effects. This limitation also affects the applicability of the results. Thirdly, due to not aggregating data from RCTs, it becomes impossible to ascertain an overall effect size for relevant parameters or determine heterogeneity between studies, which, to a certain extent, may affect the reliability of the results. The assessment of bias risk in the included RCTs revealed deficiencies in the blinding procedure, potentially contributing to placebo effects and observer bias, thereby compromising the validity of conclusions.

### 4.2 Outlook

Currently, the majority of RCTs on rTMS parameters primarily focus on comparing single parameters at different levels, which lacks a comprehensive experimental design incorporating multiple parameters and levels. Consequently, there is limited research investigating the overall efficacy of multi-parameter rTMS. In future investigations, it is crucial to employ an orthogonal experimental design method encompassing multiple parameters and levels, which can identify treatment parameter combinations that align more closely with clinical practice and provide a scientific basis for guiding clinical interventions. Furthermore, we urge researchers to prioritize blinding methodologies in their RCTs as this will enhance the credibility of research conclusions and offer valuable evidence for clinical guidance.

## 5 Conclusion

rTMS demonstrates efficacy and safety in enhancing cognitive function, quality of life, and activities of daily living among patients with post-stroke cognitive dysfunction. Furthermore, the combination of rTMS with other conventional rehabilitation methods can provide a more positive impact. However, the majority of included rTMS studies focus on single-parameter comparative analyses at varying levels, limiting our ability to determine optimal therapeutic parameters protocol for rTMS. Future research should prioritize orthogonal experimental design methods encompassing multiple parameters and levels to provide more evidence-based medical evidence into the optimal parameter combination for the clinical application of rTMS.

## Data availability statement

Original contributions from the study are included in the article and further inquiries can be directed to the corresponding author.

## Author contributions

YW: Writing – original draft, Writing – review & editing. LW: Writing – original draft. XN: Writing – original draft. MJ: Writing – original draft. LZ: Writing – review & editing.

## References

[ref1] Alevizaki M. Synetou M. Xynos K. Pappa T. Vemmos K. N. (2007). Low triiodothyronine: a strong predictor of outcome in acute stroke patients. Eur. J. Clin. Investig. 37, 651–657. doi: 10.1111/j.1365-2362.2007.01839.x, PMID: 17635576

[ref2] Boggio P. S. Rocha M. Oliveira M. O. Fecteau S. Cohen R. B. Campanhã C.. (2010). Noninvasive brain stimulation with high-frequency and low-intensity repetitive transcranial magnetic stimulation treatment for posttraumatic stress disorder. J. Clin. Psychiatry 71, 992–999. doi: 10.4088/JCP.08m04638blu, PMID: 20051219 PMC3260527

[ref3] Chen H. J. Cong S. Cheng G. Q. Dong B. Tang M. (2017). The efficacy of transcranial magnetic stimulation in treating patients with post-stroke cognitive dysfunction. Chin. J. Phys. Med. Rehab. 39, 677–679. doi: 10.3760/cma.j.issn.0254-1424.2017.09.010

[ref4] Chen Z. Y. Gong J. Q. Wu Y. F. Ping R. X. Sun Y. T. Ni X. X.. (2019). The efficacy of repetitive transcranial magnetic stimulation combined with cognitive rehabilitation training in the treatment of post-stroke cognitive impairment. Chin. J. Phys. Med. Rehab. 41, 199–201. doi: 10.3760/cma.j.issn.0254-1424.2019.03.008

[ref5] Cumpston M. Li T. Page M. J. Chandler J. Welch V. A. Higgins J. P. T.. (2019). Updated guidance for trusted systematic reviews: a new edition of the Cochrane handbook for systematic reviews of interventions. Cochrane Libr 10:142. doi: 10.1002/14651858.ed000142 PMC10284251 31643080

[ref6] Devanand D. P. van Heertum R. Kegeles L. S. Liu X. Jin Z. H. Pradhaban G.. (2010). (99m) Tc hexamethyl-propylene-aminoxime single-photon emission computed tomography prediction of conversion from mild cognitive impairment to Alzheimer disease. Am. J. Geriatric Psychiatr. 18, 959–972. doi: 10.1097/JGP.0b013e3181ec8696, PMID: 20808143 PMC3103107

[ref7] Ding Q. F. Li Z. Guo G. H. Guan C. X. Le L. Hao D. J.. (2019). Effects of repetitive transcranial magnetic stimulation with different frequencies on cognitive impairment in stroke patients. Chin. J. Rehab. 34, 513–517. doi: 10.3870/zgkf.2019.010.002

[ref8] Douiri A. Rudd A. G. Wolfe C. D. A. (2013). Prevalence of poststroke cognitive impairment: South London stroke register 1995–2010. Stroke 44, 138–145. doi: 10.1161/STROKEAHA.112.670844 23150656

[ref9] Duan L. Ding S. Y. (2016). Effect of TMS on plasma CRP and fib levels in patients with vascular cognitive dysfunction after cerebral infarction. Chin. Pract. Med. 11, 41–42. doi: 10.14163/j.cnki.11-5547/r.2016.11.025

[ref10] Duering M. Zieren N. Hervé D. Jouvent E. Reyes S. Peters N.. (2011). Strategic role of frontal white matter tracts in vascular cognitive impairment: a voxel-based lesion-symptom mapping study in CADASIL. Brain 134, 2366–2375. doi: 10.1093/brain/awr169, PMID: 21764819

[ref11] Farooq M. U. Min J. Goshgarian C. Gorelick P. B. (2017). Pharmacotherapy for vascular cognitive impairment. CNS Drugs 31, 759–776. doi: 10.1007/s40263-017-0459-3 28786085

[ref12] Gao Y. R. Guan C. X. Li Z. Guo G. H. Le L. Hao D. J. (2021). Effects of high-frequency repetitive transcranial magnetic stimulation on orientation, visual perception and ADL of stroke patients with cognitive impairment and aphasia. Chin. J. Rehab. 36, 520–523. doi: 10.3870/zgkf.2021.09.002

[ref13] Gebreegziabhere Y. Habatmu K. Mihretu A. Cella M. Alem A. (2022). Cognitive impairment in people with schizophrenia: an umbrella review. Eur. Arch. Psychiatry Clin. Neurosci. 272, 1139–1155. doi: 10.1007/s00406-022-01416-6, PMID: 35633394 PMC9508017

[ref14] Gong C. Hu H. Peng X. M. Li H. Xiao L. Liu Z.. (2023). Therapeutic effects of repetitive transcranial magnetic stimulation on cognitive impairment in stroke patients: a systematic review and meta-analysis. Front. Hum. Neurosci. 17:1177594. doi: 10.3389/fnhum.2023.1177594, PMID: 37250691 PMC10213559

[ref15] Gorelick P. B. Scuteri A. Black S. E. Decarli C. Greenberg S. M. Iadecola C.. (2011). Vascular contributions to cognitive impairment and dementia: a statement for healthcare professionals from the American Heart Association/American Stroke Association. Stroke 42, 2672–2713. doi: 10.1161/STR.0b013e3182299496, PMID: 21778438 PMC3778669

[ref16] Gu Z. T. Lu J. X. Zhang S. C. Xu R. Z. Xu S. S. (2012). Efficacy of repetitive transcranial magnetic stimulation in patients with mild cognitive dysfunction after cerebral infarction. Chin. J. Rehab. Med. 27, 964–966. doi: 10.3969/j.issn.1001-1242.2012.10.021

[ref17] He Y. G. Zhou Q. (2017). Effects of repetitive transcranial magnetic stimulation on non-dementia-type vascular cognitive dysfunction. Chin. J. Phys. Med. Rehab. 39, 464–466. doi: 10.3760/cma.j.issn.0254-1424.2017.06.018

[ref18] Hernandez-Pavon J. C. Harvey R. L. (2019). Noninvasive transcranial magnetic brain stimulation in stroke. Phys. Med. Rehab. Clin. 30, 319–335. doi: 10.1016/j.pmr.2018.12.010 30954150

[ref19] Hoy K. E. Bailey N. Michael M. Fitzgibbon B. Rogasch N. C. Saeki T.. (2016). Enhancement of working memory and task-related oscillatory activity following intermittent theta burst stimulation in healthy controls. Cereb. Cortex 26, 4563–4573. doi: 10.1093/cercor/bhv193, PMID: 26400923

[ref20] Hu L. M. Zhu Q. X. Liu Y. X. Lin Y. M. Tang N. S. Su W. H.. (2016). The therapeutic effects of supplementing rehabilitation with repetitive transcranial magnetic stimulation in treating vascular cognitive impairment but no dementia. Chin. J. Phys. Med. Rehab. 38, 278–282. doi: 10.3760/cma.j.issn.0254-1424.2016.04.009

[ref21] Huang Y. Y. Chen S. D. Leng X. Y. Kuo K. Wang Z. T. Cui M.. (2022). Post-stroke cognitive impairment: epidemiology, risk factors, and management. J. Alzheimers Dis. 86, 983–999. doi: 10.3233/JAD-215644, PMID: 35147548

[ref22] Jiang W. Tang X. Y. Yuan L. J. (2014). The clinical study of transcranial magnetic stimulation(TMS) treatment for cerebral infarction patients with mild cognitive impairment. Anhui Med. J. 35, 189–191,192. doi: 10.3969/j.issn.1000-0399.2014.02.017

[ref23] Kim B. R. Kim D. Y. Ho Chun M. Hwa Yi J. Sung Kwon J. (2010). Effect of repetitive transcranial magnetic stimulation on cognition and mood in stroke patients: a double-blind, sham-controlled trial. Am. J. Phys. Med. Rehabil. 89, 362–368. doi: 10.1097/PHM.0b013e3181d8a5b1, PMID: 20407301

[ref24] Kim J. O. Lee S. J. Pyo J. S. (2020). Effect of acetylcholinesterase inhibitors on post-stroke cognitive impairment and vascular dementia: a meta-analysis. PLoS One 15:e0227820. doi: 10.1371/journal.pone.0227820, PMID: 32032361 PMC7006920

[ref25] Kozel F. A. Johnson K. A. Nahas Z. Nakonezny P. A. Morgan P. S. Anderson B. S.. (2011). Fractional anisotropy changes after several weeks of daily left high-frequency repetitive transcranial magnetic stimulation of the prefrontal cortex to treat major depression. J. ECT 27, 5–10. doi: 10.1097/YCT.0b013e3181e6317d, PMID: 20559144 PMC2975808

[ref26] Lefaucheur J. P. Aleman A. Baeken C. Benninger D. H. Brunelin J. di Lazzaro V.. (2020). Evidence-based guidelines on the therapeutic use of repetitive transcranial magnetic stimulation (rTMS): an update (2014–2018). Clin. Neurophysiol. 131, 474–528. doi: 10.1016/j.clinph.2019.11.002, PMID: 31901449

[ref27] Levkovitz Y. Isserles M. Padberg F. Lisanby S. H. Bystritsky A. Xia G.. (2015). Efficacy and safety of deep transcranial magnetic stimulation for major depression: a prospective multicenter randomized controlled trial. World Psychiatry 14, 64–73. doi: 10.1002/wps.20199, PMID: 25655160 PMC4329899

[ref28] Li H. N. Chen Y. D. Huang M. Zhong J. Huang S. F. Huang M. L.. (2023). Effects of repetitive transcranial magnetic stimulation on cognitive function, central motor conduction time and balance ability in patients with post-stroke cognitive dysfunction. Chin. J. Rehab. 38, 140–143. doi: 10.3870/zgkf.2023.03.003

[ref29] Li J. Cui L. H. Ji Y. T. (2020a). Effect of repetitive transcranial magnetic stimulation technique combined with donepezil in treating cognitive impairment after cerebral infarction. J. Clin. Med. Pract. 24, 39–42. doi: 10.7619/jcmp.202021012

[ref30] Li J. Cui Y. Jia M. Y. (2022). Effect of repetitive transcranial magnetic stimulation in the treatment of cognitive dysfunction after ischemic stroke. Chin. Med. Herald. 19, 80–83. doi: 10.20047/j.issn1673-7210.2022.32.18

[ref31] Li J. Dong J. H. Zhang K. (2023). Therapeutic effect of rTMS on post-stroke cognitive dysfunction: an analysis using functional MRI. Chin. J. Med. Phys. 40, 872–875.

[ref32] Li Q. Liu L. S. Huo J. J. He M. (2020b). The effect of transskull magnetic stimulation combined with speech auditory feedback training on cognitive function in patients with cerebral apoplexy. Chin. J. Clini. Healthcare. 23, 660–664. doi: 10.3969/J.issn.1672-6790.2020.05.020

[ref33] Li Y. Luo H. Yu Q. Yin L. Li K. Li Y.. (2020c). Cerebral functional manipulation of repetitive transcranial magnetic stimulation in cognitive impairment patients after stroke: an fMRI study. Front. Neurol. 11:977. doi: 10.3389/fneur.2020.00977, PMID: 33013646 PMC7506052

[ref34] Li H. Ma J. Zhang J. Shi W. Y. Mei H. N. Xing Y. (2021). Repetitive transcranial magnetic stimulation (rTMS) modulates thyroid hormones level and cognition in the recovery stage of stroke patients with cognitive dysfunction. Med. Sci. Monit. 27, e931914–e931911. doi: 10.12659/MSM.931914, PMID: 34686649 PMC8549488

[ref35] Li W. Wen Q. Xie Y. H. Hu A. L. Wu Q. Wang Y. X. (2022). Improvement of poststroke cognitive impairment by intermittent theta bursts: A double-blind randomized controlled trial. Brain Behav. 12:e2569. doi: 10.1002/brb3.2569, PMID: 35484991 PMC9226849

[ref36] Li Y. M. Xu L. Yang Y. Tian J. Y. Yu Q. (2015a). Effects of repetitive transcranial magnetic stimulation on cognitive ability in patients with mild cognitive impairment after ischemic stroke. Chin. J. Rehab. Theory Pract. 21, 1128–1132. doi: 10.3969/j.issn.1006-9771.2015.10.003

[ref37] Li Y. M. Xu L. Yang Y. Tian J. Y. Yu Q. (2015b). The effects of repetitive transcranial magnetic stimulation on the cognitive ability in patients with mild cognitive impairment after ischemic stroke. Chin. J. Phys. Med. Rehab. 37, 739–742. doi: 10.3760/cma.j.issn.0254-1424.2015.010.004

[ref38] Liao L. H. Huang D. Jiang X. M. Deng X. Q. Zhou B. F. (2017). Effects of high-frequency and low-frequency repetitive transcranial magnetic stimulation on cognitive function in patients with cerebral infarction. Chin. J. Phys. Med. Rehab. 39, 56–58. doi: 10.3760/cma.j.issn.0254-1424.2017.01.014

[ref39] Liu Y. Yin M. Luo J. Huang L. Zhang S. Pan C.. (2020). Effects of transcranial magnetic stimulation on the performance of the activities of daily living and attention function after stroke: a randomized controlled trial. Clin. Rehabil. 34, 1465–1473. doi: 10.1177/0269215520946386, PMID: 32748630

[ref40] Lo J. W. Crawford J. D. Desmond D. W. Godefroy O. Jokinen H. Mahinrad S.. (2019). Profile of and risk factors for poststroke cognitive impairment in diverse ethnoregional groups. Neurology 93, e2257–e2271. doi: 10.1212/WNL.0000000000008612, PMID: 31712368 PMC6937495

[ref41] Loetscher T. Potter K. J. Wong D. das Nair R. Cochrane Stroke Group (2019). Cognitive rehabilitation for attention deficits following stroke. Cochrane Database Syst. Rev. 2019:11. doi: 10.1002/14651858.CD002842.pub3, PMID: 31706263 PMC6953353

[ref42] Lu H. Zhang T. Wen M. Sun L. (2015). Impact of repetitive transcranial magnetic stimulation on post-stroke dysmnesia and the role of BDNF Val66Met SNP. Med. Sci. Monit. 21, 761–768. doi: 10.12659/MSM.892337, PMID: 25770310 PMC4370352

[ref43] Lv M. X. Liu S. J. Wang Y. Q. Liang J. J. Li T. T. (2020). Effects of 10 Hz high-frequency repetitive transcranial magnetic stimulation combined with hyperbaric oxygen on cognitive dysfunction and cerebral metabolism after stroke. Chin. J. Microcircul. 30, 26–31. doi: 10.3969/j.issn.1005-1740.2020.04.006

[ref44] Ma J. Li H. Zhang J. Zhao Q. Q. Shi W. Y. (2021). Effects of low-frequency repetitive transcranial magnetic stimulation combined with cognitive training on thyroid hormone levels and cognitive function in patients with cognitive impairment after cerebral stroke. Hebei Med. J. 43, 2436–2441. doi: 10.3969/j.issn.1002-7386.2021.16.007

[ref45] Ma J. Zhang Z. Su Y. Kang L. Geng D. Wang Y.. (2013). Magnetic stimulation modulates structural synaptic plasticity and regulates BDNF–TrkB signal pathway in cultured hippocampal neurons. Neurochem. Int. 62, 84–91. doi: 10.1016/j.neuint.2012.11.010, PMID: 23201339

[ref46] Mao J. Hong Y. F. Feng X. J. Tang X. X. Zhang J. N. Kan X. L. (2022). Effect of repetitive transcranial magnetic stimulation of different frequencies on the cognition and movement of stroke patients. Chin. J. Gen. Pract. 20, 1036–1040. doi: 10.16766/j.cnki.issn.1674-4152.002518

[ref47] Merriman N. A. Sexton E. McCabe G. Walsh M. E. Rohde D. Gorman A.. (2019). Addressing cognitive impairment following stroke: systematic review and meta-analysis of non-randomised controlled studies of psychological interventions. BMJ Open 9:e024429. doi: 10.1136/bmjopen-2018-024429, PMID: 30819706 PMC6398645

[ref48] Naghavi M. Abajobir A. A. Abbafati C. Abbas K. M. Abd-Allah F. Abera S. F.. (2017). Global, regional, and national age-sex specific mortality for 264 causes of death, 1980–2016: a systematic analysis for the global burden of disease study 2016. Lancet 390, 1151–1210. doi: 10.1016/S0140-6736(17)32152-9, PMID: 28919116 PMC5605883

[ref49] Nowak D. A. Bösl K. Podubeckà J. Carey J. R. (2010). Noninvasive brain stimulation and motor recovery after stroke. Restor. Neurol. Neurosci. 28, 531–544. doi: 10.3233/RNN-2010-0552 20714076

[ref50] Ogiue-Ikeda M. Kawato S. Ueno S. (2005). Acquisition of ischemic tolerance by repetitive transcranial magnetic stimulation in the rat hippocampus. Brain Res. 1037, 7–11. doi: 10.1016/j.brainres.2004.10.063, PMID: 15777747

[ref51] Park I. S. Yoon J. G. (2015). The effect of computer-assisted cognitive rehabilitation and repetitive transcranial magnetic stimulation on cognitive function for stroke patients. J. Phys. Ther. Sci. 27, 773–776. doi: 10.1589/jpts.27.773, PMID: 25931728 PMC4395712

[ref52] Pei S. Wang J. Xia J. Y. (2022). Effect of repetitive transcranial magnetic intermittent θ burst stimulation on post-stroke cognitive impairment. Chongqing Med. 51, 3120–3125. doi: 10.3969/j.issn.1671-8348.2022.18.012

[ref53] Qi F. Su Y. L. Zhang H. (2021). Efficacy of hyperbaric oxygen combined with repetitive transcranial magnetic stimulation on patients with cognitive impairment after cerebral infarction and its effect on hemodynamics. Chin. J. Naut. Med. Hyperbaric Med. 28, 383–388. doi: 10.3760/cma.j.cn311847-20201117-00426

[ref54] Ren Y. Gu X. D. Yao Y. H. Fu J. M. Yin H. K. Li L.. (2018). The effect of hyperbaric oxygen therapy combined with repetitive transcranial magnetic stimulation on patients with cognitive dysfunction after cerebral infarction. Chin. J. Phys. Med. Rehab. 40, 336–339. doi: 10.3760/cma.j.issn.0254-1424.2018.05.004

[ref55] Rohde D. Gaynor E. Large M. Mellon L. Hall P. Brewer L.. (2019). The impact of cognitive impairment on poststroke outcomes: A 5-year follow-up. J. Geriatr. Psychiatry Neurol. 32, 275–281. doi: 10.1177/0891988719853044, PMID: 31167593

[ref56] Rossi S. Hallett M. Rossini P. M. Pascual-Leone A. Safety of TMS Consensus Group (2009). Safety, ethical considerations, and application guidelines for the use of transcranial magnetic stimulation in clinical practice and research. Clin. Neurophysiol. 120, 2008–2039. doi: 10.1016/j.clinph.2009.08.016, PMID: 19833552 PMC3260536

[ref57] Selimbeyoglu A. Parvizi J. (2010). Electrical stimulation of the human brain: perceptual and behavioral phenomena reported in the old and new literature. Front. Hum. Neurosci. 4:46. doi: 10.3389/fnhum.2010.00046, PMID: 20577584 PMC2889679

[ref58] Seniów J. Waldowski K. Leśniak M. Iwański S. Czepiel W. Członkowska A. (2013). Transcranial magnetic stimulation combined with speech and language training in early aphasia rehabilitation: a randomized double-blind controlled pilot study. Top. Stroke Rehabil. 20, 250–261. doi: 10.1310/tsr2003-250, PMID: 23841973

[ref59] Sun M.-K. (2018). Potential therapeutics for vascular cognitive impairment and dementia. Curr. Neuropharmacol. 16, 1036–1044. doi: 10.2174/1570159X15666171016164734, PMID: 29046153 PMC6120112

[ref60] Sun W. Mao W. Meng X. Wang D. Qiao L. Tao W.. (2012). Low-frequency repetitive transcranial magnetic stimulation for the treatment of refractory partial epilepsy: a controlled clinical study. Epilepsia 53, 1782–1789. doi: 10.1111/j.1528-1167.2012.03626.x, PMID: 22950513

[ref61] Tang X. Y. Yuan L. J. Jiang M. K. Chen Z. S. (2015). Effects of repetitive transcranial magnetic stimulation on cognitive function in post-cerebral infarction patients with mild cognitive impairment. Stroke Nerv. Dis. 22, 76–79. doi: 10.3969/j.issn.1007-0478.2015.02.003

[ref62] Trung J. Hanganu A. Jobert S. Degroot C. Mejia-Constain B. Kibreab M.. (2019). Transcranial magnetic stimulation improves cognition over time in Parkinson’s disease. Parkinsonism Relat. Disord. 66, 3–8. doi: 10.1016/j.parkreldis.2019.07.006, PMID: 31300260

[ref63] Tsai P. Y. Lin W. S. Tsai K. T. Kuo C. Y. Lin P. H. (2020). High-frequency versus theta burst transcranial magnetic stimulation for the treatment of poststroke cognitive impairment in humans. J. Psychiatry Neurosci. 45, 262–270. doi: 10.1503/jpn.190060, PMID: 32159313 PMC7828923

[ref64] Tsao C. W. Aday A. W. Almarzooq Z. I. Anderson C. A. M. Arora P. Avery C. L.. (2023). Heart disease and stroke statistics—2023 update: a report from the American Heart Association. Circulation 147, e93–e621. doi: 10.1161/CIR.0000000000001123, PMID: 36695182 PMC12135016

[ref65] Valero-Cabre A. Pascual-Leone A. Rushmore R. J. (2008). Cumulative sessions of repetitive transcranial magnetic stimulation (rTMS) build up facilitation to subsequent TMS-mediated behavioural disruptions. Eur. J. Neurosci. 27, 765–774. doi: 10.1111/j.1460-9568.2008.06045.x, PMID: 18279329

[ref66] Van den Boom M. A. Jansma J. M. Ramsey N. F. (2018). Rapid acquisition of dynamic control over DLPFC using real-time fMRI feedback. Eur. Neuropsychopharmacol. 28, 1194–1205. doi: 10.1016/j.euroneuro.2018.08.508, PMID: 30217551 PMC6420021

[ref67] Vos T. Lim S. S. Abbafati C. Abbas K. M. Abbasi M. Abbasifard M.. (2020). Global burden of 369 diseases and injuries in 204 countries and territories, 1990–2019: a systematic analysis for the global burden of disease study 2019. Lancet 396, 1204–1222. doi: 10.1016/S0140-6736(20)30925-9, PMID: 33069326 PMC7567026

[ref68] Wang K. Dong Q. Yu J. T. Hu P. P. (2021a). Expert Consensus on post-stroke cognitive impairment management 2021. Chin. J. Stroke. 16, 376–389. doi: 10.3969/j.issn.1673-5765.2021.04.011

[ref69] Wang S. Y. Gu Z. K. Chen W. Wang M. Bi Y. L. (2021b). Effects of repetitive transcranial magnetic stimulation at different frequencies on cognitive impairment after stroke. Chin. J. Phys. Med. Rehab. 43, 721–723. doi: 10.3760/cma.j.issn.0254-1424.2021.08.012

[ref70] Wu S. P. Ji X. Qi Y. W. Wang H. Ma J. J. (2022). Combining transcranial magnetic stimulation with action observation therapy better improves the neurological functioning of stroke survivors. Chin. J. Phys. Med. Rehab. 44, 35–39. doi: 10.3760/cma.j.issn.0254-1424.2022.01.006

[ref71] Xu B. Y. Gu Z. K. Wang S. Y. Wang X. Ma Z. Z. (2022). Effects of high-frequency repetitive transcranial magnetic stimulation combined with speech-auditory feedback training on event-related potential P300 and serum NSE and S100β proteins in patients with post-stroke cognitive impairment. Prog. Mod. Biomed. 22, 4541–4545. doi: 10.13241/j.cnki.pmb.2022.23.028

[ref72] Yin M. Y. Luo J. Hu X. Q. Xian Q. L. Huang L. Zhang S. X.. (2018). Effects of high-frequency repetitive transcranial magnetic stimulation on post-stroke cognitive impairment. Chin. J. Phys. Med. Rehab. 33, 763–769. doi: 10.3969/j.issn.1001-1242.2018.07.003

[ref73] Yin Y. K. Wang G. L. Sun J. Z. (2023). Therapeutic effect of different-frequency repetitive transcranial magnetic stimulations on post-stroke cognitive impairment: a Meta-analysis. Chin. J. Tiss. Eng. Res. 27, 3274–3280. doi: 10.12307/2023.150

[ref74] Yingli B. Zunke G. Wei C. Shiyan W. (2022). Cerebral activity manipulation of low-frequency repetitive transcranial magnetic stimulation in post-stroke patients with cognitive impairment. Front. Neurol. 13:951209. doi: 10.3389/fneur.2022.951209, PMID: 36425802 PMC9679635

[ref75] You F. Tian M. Jiang Z. W. Qian J. Y. Chen B. (2023). Effects of enriched rehabilitation training combined with rTMS on cognitive function, plasma miR-146a-5p and TNF-α levels in patients with post-stroke cognitive impairment. Chin. J. Pract. Nerv. Dis. 26, 842–847. doi: 10.12083/SYSJ.221489

[ref76] Yu Z. Y. Zhang W. M. (2019). Effects of repetitive transcranial magnetic stimulation on patients with cognitive impairment. Rehab. Med. 29, 20–26. doi: 10.3724/SP.J.1329.2019.05020

[ref77] Zhang J. Ma J. Li H. Mei H. N. Tao X. L. (2021). Effects of repetitive transcranial magnetic stimulation on post-stroke cognitive impairment and lipid metabolism. Chinese. J. Rehabil. 36, 584–588. doi: 10.3870/zgkf.2021.10.002

[ref78] Zhang J. J. Wu L. L. Chen D. D. Ren J. X. Liu L. Li Y. (2022). The effects of high-frequency repetitive transcranial magnetic stimulation on cognitive function after stroke observed based on electroencephalogram nonlinear analysis. J. Chongqing Med. Univ. 47, 762–767. doi: 10.13406/j.cnki.cyxb.003059

[ref79] Zhang F. Zhou S. Y. (2019). Effects of high frequency repetitive transcranial magnetic stimulation on cognitive function in stroke patients in convalescent stage. Chin. J. Pract. Nerv. Dis. 22, 2479–2485. doi: 10.12083/SYSJ.2019.22.404

[ref80] Zhang J. J. Zhu M. L. Hu D. X. Jiang Y. Liu B. L. Wang Y. J.. (2020). The efficacy of different frequencies of repetitive transcranial magnetic stimulation in the treatment of mild cognitive impairment after cerebral infarction. Shandong Med. J. 60, 59–62. doi: 10.3969/j.issn.1002-266X.2020.10.015

[ref81] Zhao X. X. Han X. H. Zhang J. H. Huang X. L. (2012). Effects of high-frequency repetitive transcranial magnetic stimulation on learning and memory of rats with cerebral infarction. Chin. J. Rehab. Med. 27, 1087–1092. doi: 10.3969/j.issn.1001-1242.2012.12.001

[ref82] Zheng C. J. Xia W. G. Duan C. Li Z. L. Wang J. Cui X. Y.. (2020). Repeated transcranial magnetic stimulation combined with donepezil can improve the cognition of cognitively impaired stroke survivors. Chin. J. Phys. Med. Rehab. 42, 32–36. doi: 10.3760/cma.j.issn.0254-1424.2020.01.008

[ref83] Zhou T. Gu Z. K. Wang S. Y. Wang M. Wang Y. (2017). Effects of repetitive transcranial magnetic stimulation on post stroke cognitive impairment. J. Xuzhou Med. Univ. 37, 108–111. doi: 10.3969/j.issn.1000-2065.2017.02.012

[ref84] Zuo J. J. Zhang H. (2013). Effect of transcranial magnetic stimulation on cognitive impairment, C-reactive protein and fibrinogen in ischemic stroke patients. Chin. J. Geriatric Heart Brain Vessel Dis. 15, 614–616. doi: 10.3969/j.issn.1009-0126.2013.06.017

