# Supplementary material for: Efficacy of repetitive transcranial magnetic stimulation with different application parameters for post-stroke cognitive impairment: a systematic review
Source: Front Neurosci. 2024 Mar 19;18:1309736. doi: 10.3389/fnins.2024.1309736 (PMC10985147; doi:10.3389/fnins.2024.1309736)
Supplement: Supplementary file 1 [file Data_Sheet_1.docx]

***Supplementary materials***

**Efficacy of repetitive transcranial magnetic stimulation with different application parameters for post-stroke cognitive impairment: a systematic review**

**Yuhan Wang^1^, Linjia Wang^1^, Xixiu Ni^1^,Minjiao Jiang^2^, Ling Zhao^1＊^**

**^＊^Correspondence:**

Corresponding: Ling Zhao

zhaoling@cdutcm.edu.cn

**1. Supplementary tables**

**Table 1 Search Strategy**

| **NO.** | **Search Items** |
| --- | --- |
| **#1** | Transcranial Magnetic Stimulation OR Repetitive Transcranial Magnetic Stimulation OR Repeated transcranial magnetic stimulation OR TMS OR rTMS OR theta burst stimulation OR TBS[Title/Abstract] |
| **#2** | Stroke OR Cerebrovascular Accident OR CVA (Cerebrovascular Accident) OR Cerebrovascular Apoplexy OR Brain Vascular Accident OR Cerebrovascular Stroke OR Apoplexy OR Cerebral Stroke OR Acute Stroke OR Acute Cerebrovascular Accident OR cerebral hemorrhage OR intracerebral hemorrhage OR cerebral infarction OR brain infarction OR acute cerebral infarction[Title/Abstract] |
| **#3** | Cognitive Dysfunction OR Cognitive Impairment OR Cognitive Disorder OR Mild Cognitive Impairment OR Cognitive Decline OR Mental Deterioration OR Cognitive Function [Title/Abstract] |
| **#4** | #1 AND #2 AND #3 |

**Table 2 Search Strategy**

| **NO.** | **Search Items** |
| --- | --- |
| **#1** | 经颅磁刺激 OR重复经颅磁刺激 OR θ脉冲刺激 OR θ爆发刺激 OR θ节律刺激 OR Theta脉冲刺激 OR Theta爆发刺激 OR Theta节律刺激OR rTMS OR TMS OR TBS[主题] |
| **#2** | 中风 OR 卒中 OR 脑卒中 OR 脑梗死 OR 脑出血 OR出血性卒中 OR 出血性中风 OR 脑血管意外[主题] |
| **#3** | 认知障碍 OR 认知缺损 OR 认知残疾 OR 痴呆 OR 呆病 OR 认知减退 OR 认知下降 OR 认知功能[主题] |
| **#4** | #1 AND #2 AND #3 |

**2. Supplementary Figures**

**
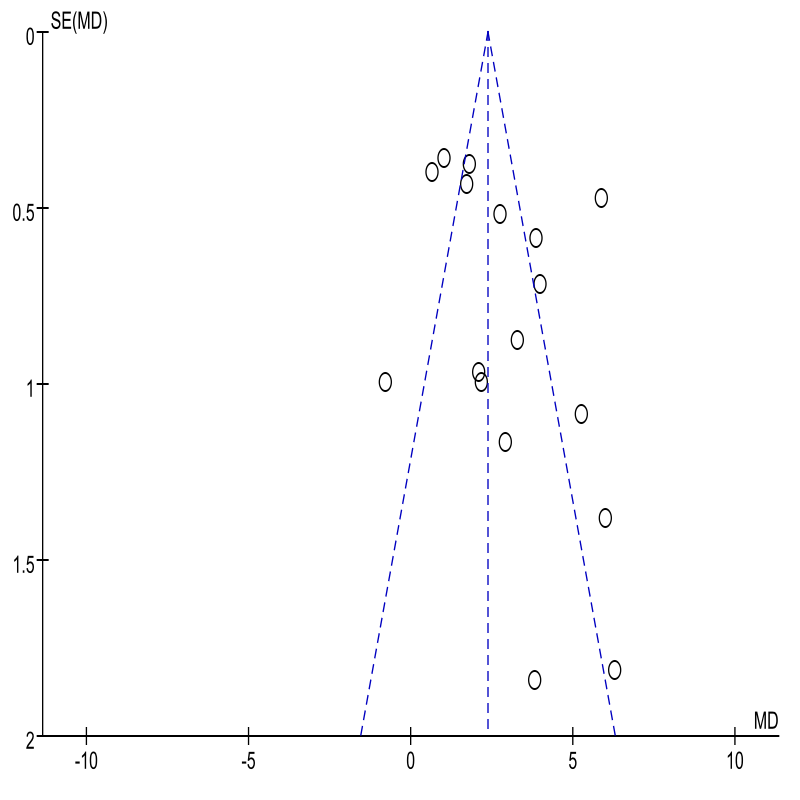
**

**Figure 1 Mini-Mental State Examination**


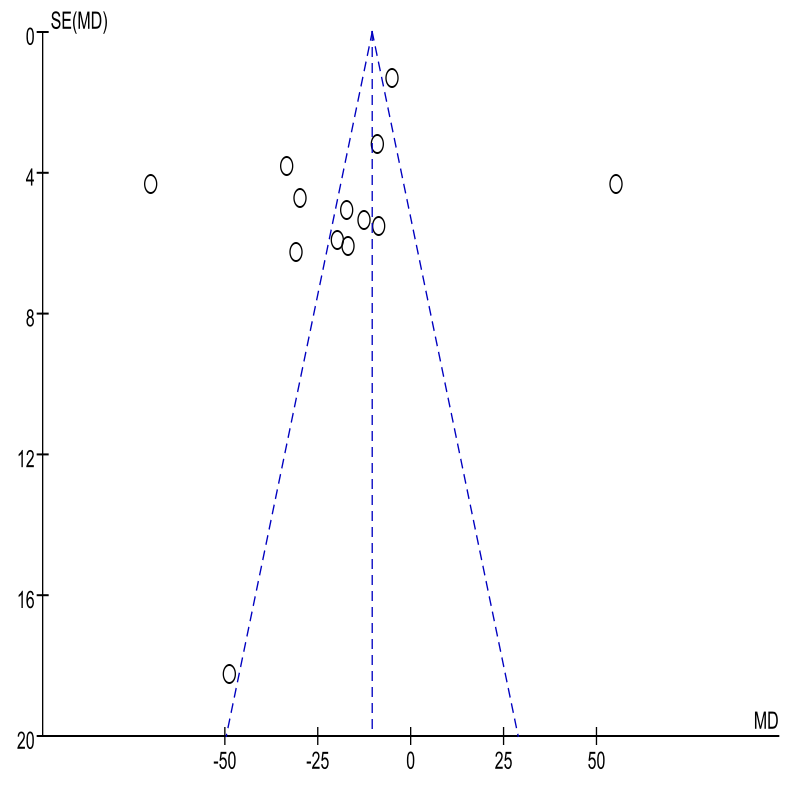


**Figure 2 Auditory Event-Related Potential P3000 Latency**


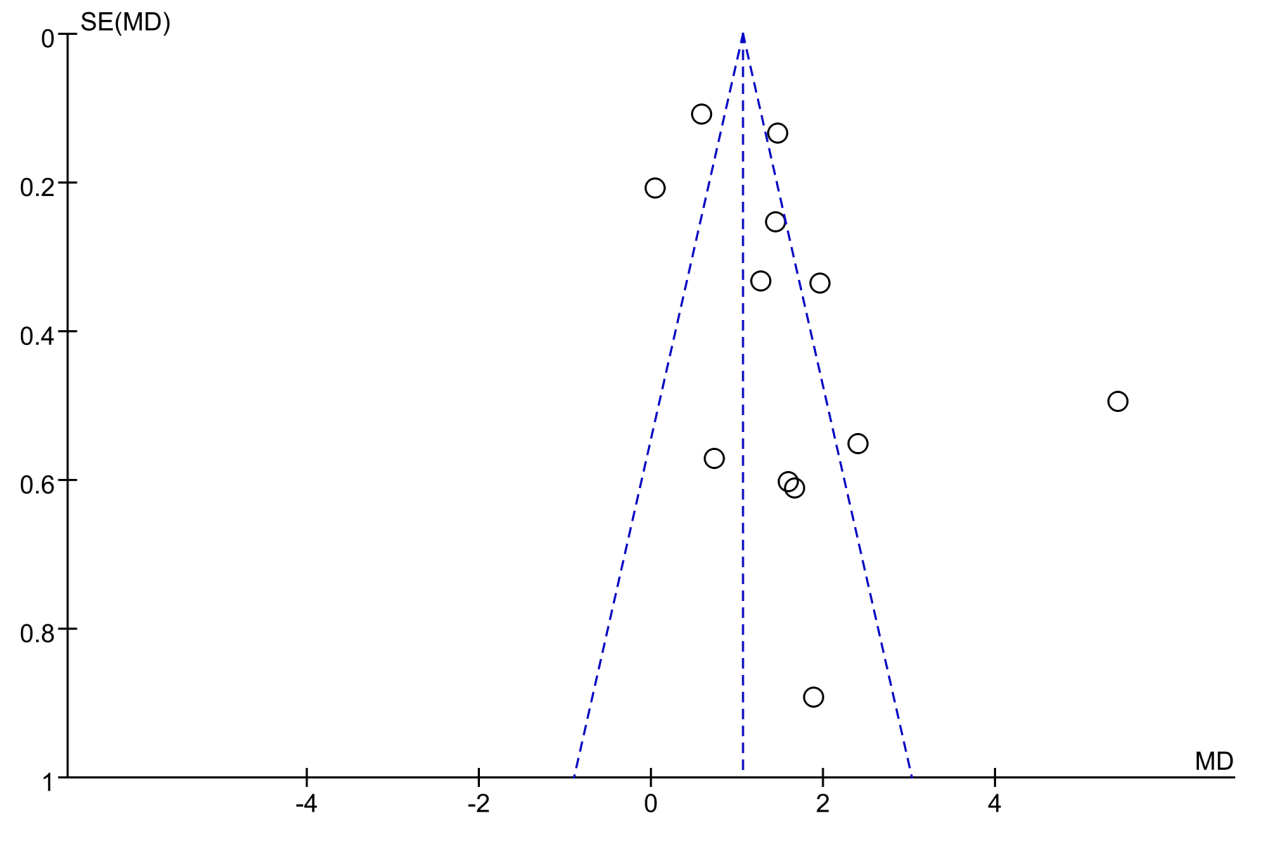


**Figure 3 Auditory Event-Related Potential P3000 Amplitude**

**
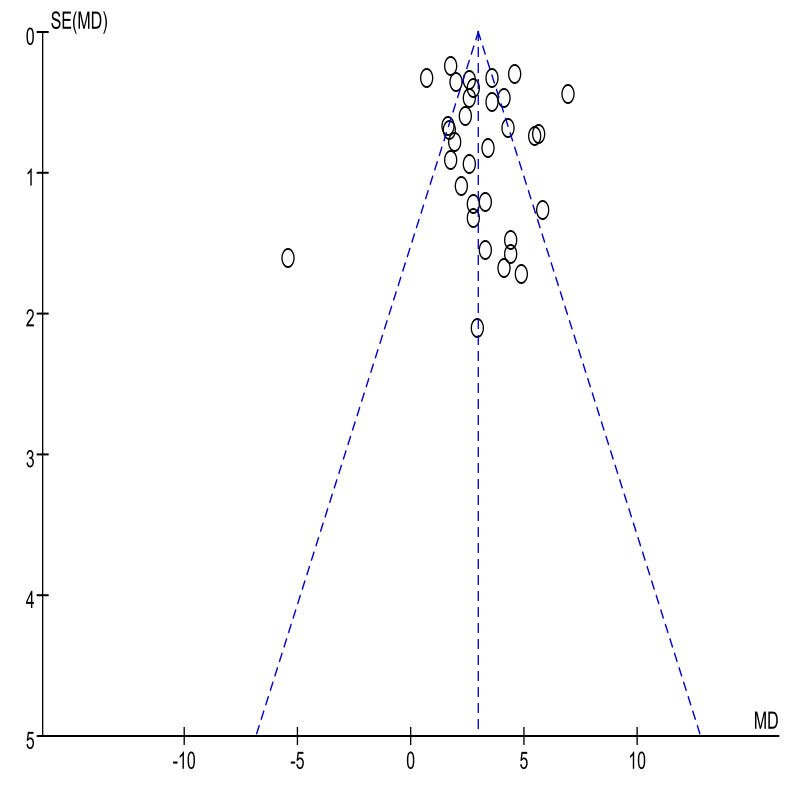
**

**Figure 4 Montreal Cognitive Assessment Scale**
